# Draft genome sequence and metabolomics data for *Streptomyces* sp. ADLamb9 isolated from the rhizosphere of *Lavandula dentata*

**DOI:** 10.1016/j.dib.2025.112199

**Published:** 2025-10-24

**Authors:** Adline Dely, Riccardo Racicot, Robert Samples, Lesley-Ann Giddings

**Affiliations:** a Department of Chemistry, Smith College, Northampton, Massachusetts, 01063, USA; b Biochemistry Program, Smith College, Northampton, Massachusetts, 01063, USA

**Keywords:** Siderophores, Iron acquisition, Microbial ecology, Actinobacteria

## Abstract

Iron-chelating molecules or siderophores play pivotal roles in soil ecosystems, particularly in facilitating plant iron uptake as well as the phytoremediation of metal-polluted environments. *Lavandula dentata*, commonly referred to as French Lavender, is a valuable species for siderophore production due to its ability to thrive in iron-deficient Mediterranean soils by forming symbiotic relationships with siderophore-producing rhizosphere microbes. Here, we used a Chrome Azurol S (CAS) overlay assay to isolate a yellow-pigmented *L. dentata* rhizosphere siderophore-producing bacterium. This isolate also demonstrated antibacterial and antifungal activities against *Bacillus subtilis* and *Aspergillus flavus,* respectively. Genomic sequencing revealed that the isolate was *Streptomyces* sp. ADLamb9 with a genome size of 8.2 Mb and 71.77% GC content. antiSMASH analysis of the *Streptomyces* sp. ADLamb9 genome identified four putative siderophore biosynthetic gene clusters as well as the catecholate siderophore mirubactin. Liquid chromatography-tandem mass spectrometry (LC-MS/MS) masses consistent with desferrioxamine B (561.3604 *m/z*), IC202C (517.3342 *m/z*), mirubactin (605.2207 *m/z*), as well as previously unreported desferrioxamine A_1C_. Notably, the presence of the rare earth element cerium differentially affected the accumulation of catecholate and hydroxamate siderophores, highlighting our incomplete understanding of the complex regulation and relationship between siderophore biosynthesis genes. These datasets, deposited at NCBI under the BioProject accession number PRJNA1224804, contribute to the broader scientific understanding of metabolite diversity and genomic features of *Streptomyces* sp. ADLamb9, providing insight into its use in bioremediation, especially in the presence of rare earth elements.

Specifications Table

**Table:** 

Subject	Ecology
Specific subject area	MultiOmics
Type of data	Raw and processed data (tables, figures, images, mass spectra)
Data collection	*Streptomyces* sp. ADLamb9 was isolated from soil collected from the Show House of the Botanic Garden at Smith College. Bacterial extracts were analyzed by liquid chromatography/mass spectrometry (LC/MS) (Q-exactive, Thermo Scientific). Raw LC/MS data were processed using MS-DIAL v5.5.241113 with default settings, including profile data for both MS1 and MS2 scans, and selection of [M+H] and [M+Na] adducts. A minimum peak intensity threshold of 3e5 was set, and the peak lists were analyzed with MPACT v1.00 r24.11.17. The data were filtered and visualized using the default settings in MPACT. To confirm siderophore production, the Chrome Azurol S agar assay was performed. Antibacterial and antifungal activities were assessed against *Bacillus subtilis* and *Aspergillus flavus* on GYM (glucose, yeast extract, and malt extract) agar. DNA extraction from selected isolates was carried out using the ZymoBIOMICS DNA Microprep Kit (Zymo Research, Irvine, CA). Genomic DNA was sequenced using the Illumina MiSeq platform with the MiSeq Reagent Kit v3 (600 cycles). Assembly of the genomic data was performed using Quast v5.2.0, BLAST, and Unicycler v0.5.0 (via Galaxy). Phylogenetic analysis of 16S rRNA was performed by TYGS and further confirmed by Sanger sequencing. The genome was also annotated by RAST. Secondary metabolite biosynthetic gene clusters (BGCs) were predicted using antiSMASH v6.0.0.
Data source location	The Show House of the Botanic Garden at Smith College (42°19′05.7"N 72°38′23.5"W) City/Town/Region: Northampton, MA, 01063 Country: United States
Data accessibility	Sequencing data were deposited in the National Center for Biotechnology Information (NCBI) GenBank database under the accession number PRJNA1224804. Liquid chromatography-mass spectrometry (LC-MS) .raw files and 16S rDNA sequence are available in FigShare, a public repository. Data identification number: 10.6084/m9.figshare.29649722
Related research article	None

## 1 Value of the Data

• The draft genome sequence and metabolomics data contribute to understanding the biosynthesis of bioactive secondary metabolites from *L. dentata-*associated rhizosphere bacteria.
• Chemists and biochemists studying siderophores and their metal-binding properties can build on this data to investigate new siderophores, siderophore regulatory mechanisms, or optimize siderophore production for applications in medicine, such as iron chelation therapies.
• These multi-Omics data could be utilized by researchers exploring natural antimicrobial compounds, as siderophores often play dual roles in microbial competition and pathogen suppression.
• These data can be applied to phytoremediation research, where siderophore-producing bacteria may enhance the removal of heavy metals from contaminated environments through plant-microbe interactions.
• Metabolomics of *Streptomyces* grown in the presence of rare earth elements, such as cerium, provides insight into their impact on bacterial secondary metabolism and demonstrates ways in which these elicitors can be exploited to access new chemical space.

## 2 Background

Aromatic plants, such as *Lavandula dentata*, demonstrate significant potential in cleaning up metal-polluted areas by reducing metal mobility, hyperaccumulating heavy metals, and bio-monitoring soil quality [1]. Siderophores play a pivotal role in their iron acquisition by facilitating the uptake of highly soluble ferrous iron and insoluble ferric iron [2]. While siderophore activity in the *L. dentata* rhizosphere has been reported, their biosynthetic gene clusters (BGCs) have not been explored to better understand their roles in iron acquisition [3]. This dataset investigates the genes and secondary metabolites involved in siderophore biosynthesis within an *L. dentata* rhizosphere bacterium*. L. dentata* soil samples were cultured on GYM (glucose, yeast extract, and malt extract) and marine agar to isolate actinobacteria, which are known producers of secondary metabolites, especially siderophores [2]. Marine agar was used to mimic the saline osmotic conditions typical of the coastal Mediterranean habitat of *L. dentata* [3]. Siderophore production was assessed using a colorimetric Chrome azurol S (CAS) overlay assay as well as liquid chromatography-mass spectrometry [1]. Cerium, a rare earth element known to modulate secondary metabolite production in some soil bacteria, was also used to investigate siderophore production [4]. This dataset contributes to a better understanding of siderophore production and the use of *L. dentata* rhizosphere bacteria for bioremediation.

## 3 Data Description

**Fig. 1:**
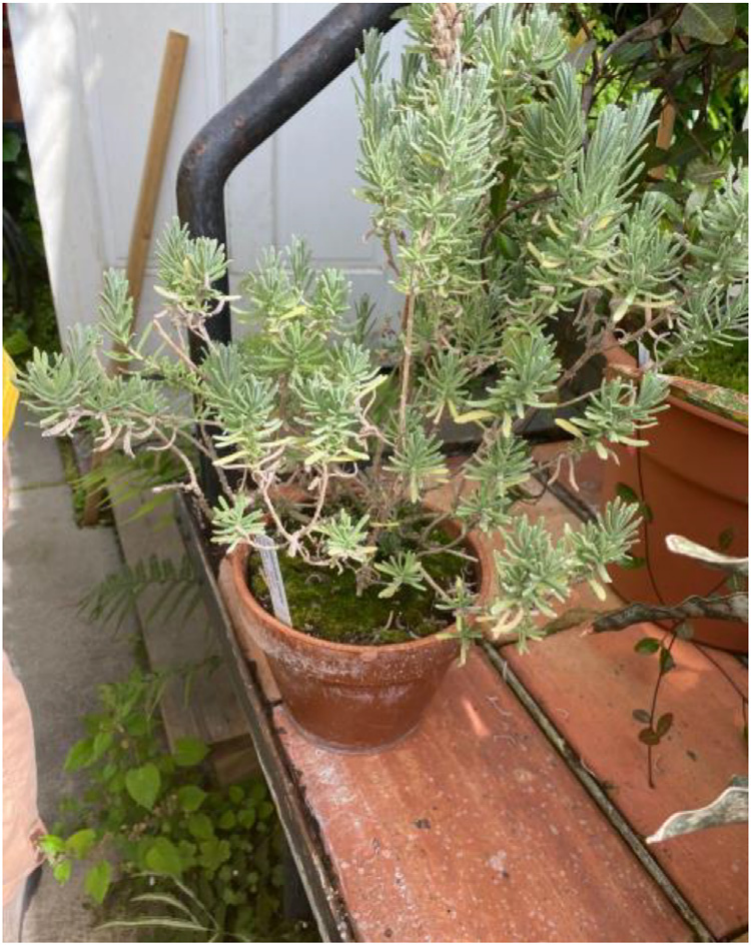
*Lavandula dentata* ‘Lambikins’ within the Lamiaceae family, located in the Show House of the Botanic Garden at Smith College.

**Fig. 2:**
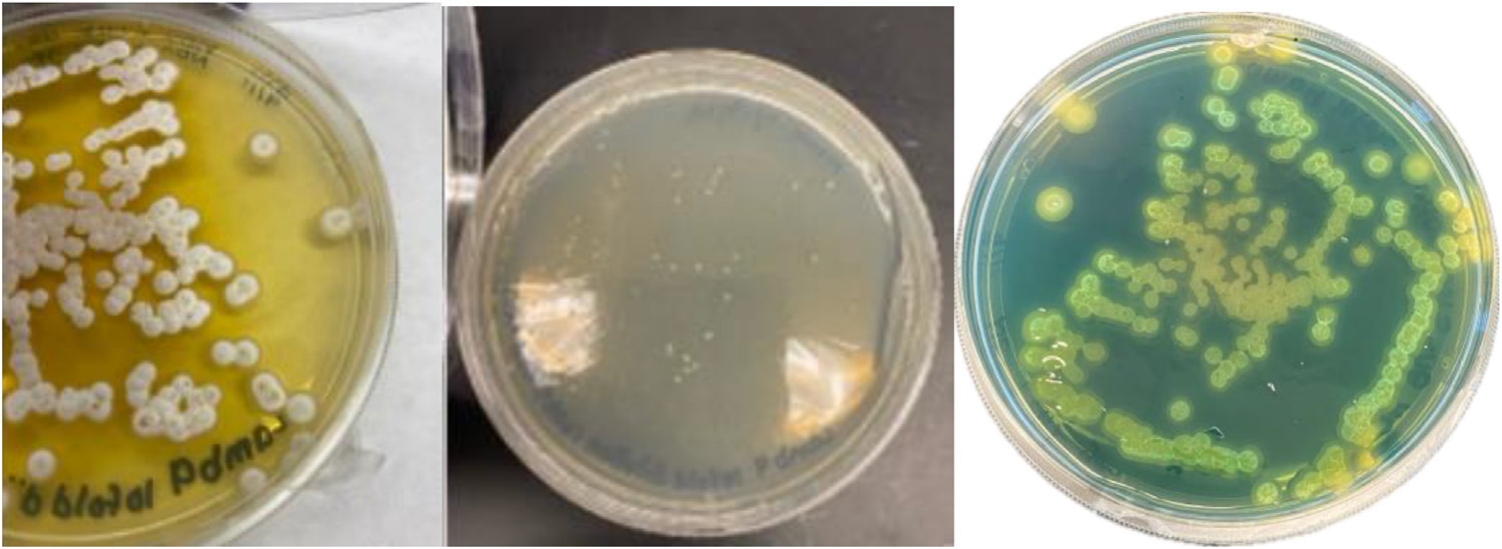
*Streptomyces* sp. ADLamb9 on GYM agar (left), marine agar (center), and CAS overlay assay (right). Orange halos on CAS overlay appeared after one week, indicating siderophore production.

**Fig. 3:**
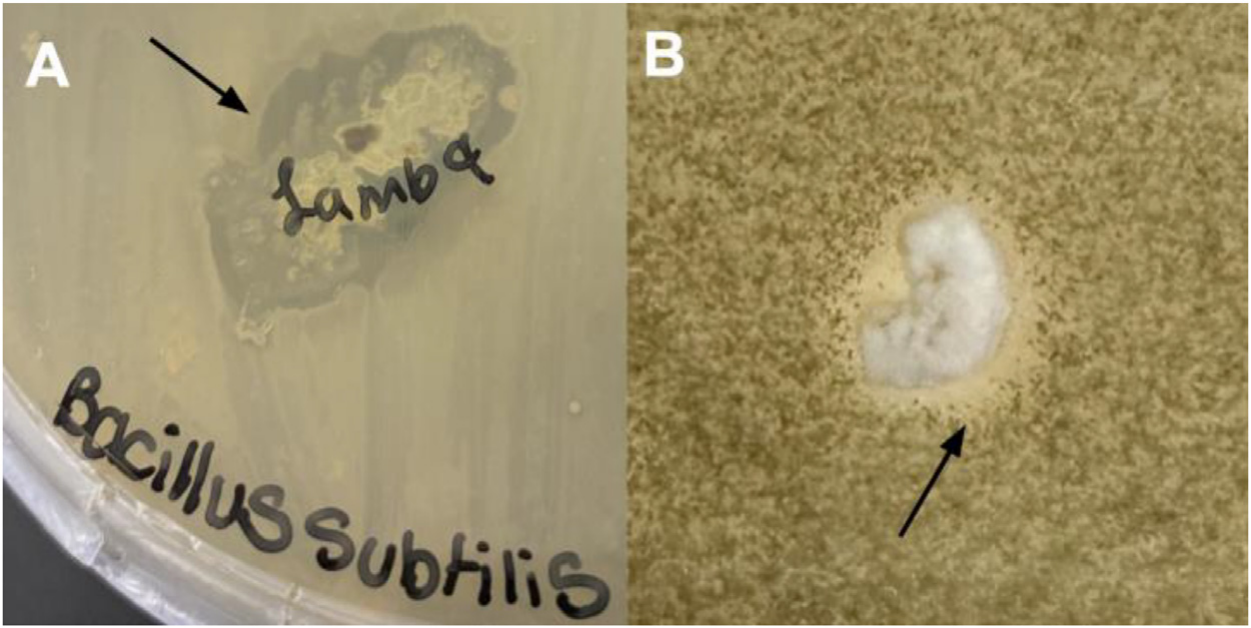
Images of zones of inhibition for *Streptomyces* sp. ADLamb9 against *B. subtilis* (A) and *A. flavus* (B) on GYM agar. Black arrows denote zones.

*Streptomyces* sp. ADLamb9 was isolated from the rhizosphere of *L. dentata* (Fig. 1) obtained from the Show House of The Botanic Garden at Smith College (42°19′05.7``N 72°38′23.5''W). The *Streptomyces* sp. ADLamb9 isolate was cultured on either GYM (glucose, yeast extract, and malt extract) agar containing nalidixic acid and cycloheximide or marine agar to assess its environmental preferences and growth rates. The isolated bacterium was yellow-pigmented, with a moderately sized, rounded colony with a smooth, convex surface and an entire margin, displaying a white fuzzy appearance, as shown in Fig. 2. On GYM agar, *Streptomyces* sp. ADLamb9 showed robust growth (Fig. 2), whereas growth was limited on marine agar. Due to its similarity to actinobacteria, *Streptomyces* sp. ADLamb9 was evaluated for siderophore activity using a CAS overlay assay and produced a positive reaction, resulting in orange halos (Fig. 2). Furthermore, *Streptomyces* sp. ADLamb9 exhibited antibacterial and antifungal activity against *Bacillus subtilis* (1) and *Aspergillus flavus* (2) (Fig. 3). Fig. 3 shows representative images showing clear inhibition zones.

We further explored siderophore biosynthesis in this isolate by sequencing its genome. The draft genome of *Streptomyces* sp. ADLamb9 (NCBI BioProject PRJNA1224804) had a total length of 8,235,241 bp with 315 contigs and GC content of 71.77%. The total number of reads was 1,590,042, and the total number of reads passing QC was 1,567,403. The N50 value was 40,224 bp and there are no ambiguous bases (# N’s = 0). The estimated genome completeness was 98%, and no foreign contamination was detected [5,6]. Approximately 98% of single copy genes from the genus *Streptomyces* were present in the genome [5] (Table 1).

**Table 1:** Assessment of the quality of the assembly through QUAST (11.6.2024).

Statistics without reference	
# contigs	315
# contigs (≥ 0 bp)	320
# contigs (≥ 1000 bp)	308
Largest contig	202,490
Total length	8,235,241
Total length (≥ 0 bp)	8,236,418
Total length (≥ 1000 bp)	8,229,797
GC (%)	71.77
N50	40,224
# N's	0

**Fig. 4:**
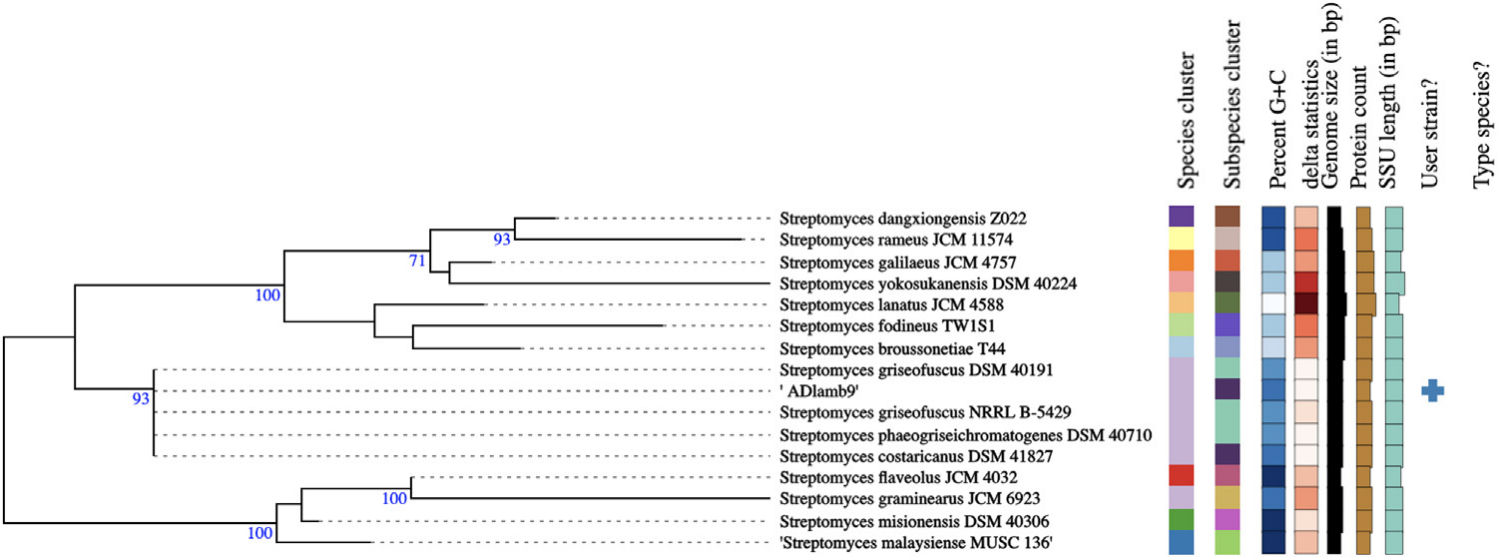
TYGS 16S rDNA gene analysis of *Streptomyces* sp. ADLamb9 and its closely related type strain [8]. The phylogenetic tree was inferred with FastME 2.1.4 [9] from Genome BLAST Distance Phylogeny (GBDP) based distances calculated from 16S rDNA gene sequences and rooted at the midpoint [8]. The branch lengths are scaled in terms of the GBDP distance formula *d_5_*. The numbers above branches are GBDP pseudo-bootstrap support values >60% from 100 replications, with an average branch support of 59.7% [8,9].

Comparison of the whole genome and partial 16S ribosomal RNA sequence (1427 nucleotides, >98% sequence identity and 100% query coverage) to NCBI genomic databases also revealed that the isolated bacterium was a strain of *Streptomyces* [7]. Further taxonomic analysis was performed via digital DNA-DNA hybridization (DDH) using the Type Strain Genome Server (TYGS) platform [8]. The closest related species was identified as *Streptomyces costaricanus* DSN 41827 with 91.6% identity. A phylogenetic tree was reconstructed using 16S rDNA gene sequence data from *Streptomyces* sp. ADLamb9 and closely related type strain using the TYGS platform, which revealed this isolate is closely related to various strains of *Streptomyces* (Fig. 4) [8].

**Fig. 5:**
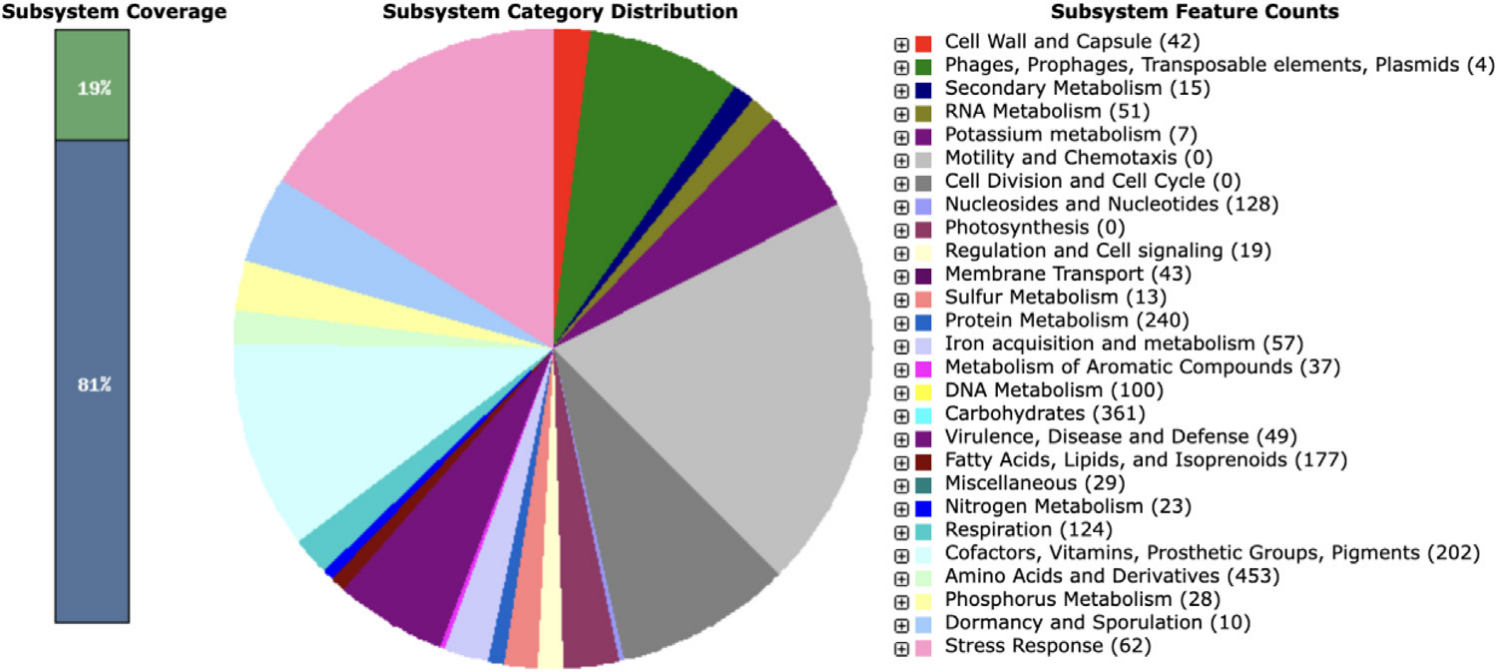
Subsystem statistics information of *Streptomyces* sp. ADLamb9 using RAST annotation. The subsystems category and corresponding feature counts are shown in the legend.

Genes were annotated and grouped with others involved in similar processes, functions, or subsystems. RAST annotation of the genome detected 316 contigs with protein-coding genes, 324 subsystems with 7657 coding sequences, and 71 RNAs (Fig. 5) [10]. Fifteen genes were annotated as being part of iron acquisition and metabolism, and 57 were associated with secondary metabolism. antiSMASH analyses also identified 39 BGCs involved in producing secondary metabolites, including at least four involved in siderophore biosynthesis (Table 2) [11]. Two BGCs are highly similar to genes involved in the production of desferrioxamines and mirubactin.

**Table 2:** Putative gene clusters predicted in the genome of ADLamb9 by antiSMASH 6.1.1. (11.6.2024). NRPS, nonribosomal peptide synthase; RRE-containing, RiPP recognition element-containing; transAT-PKS-like, trans-acyltransferase-polyketide synthase-like; T1PKS, type-1 polyketide synthase; T2PKS, type-2 polyketide synthase; and T3PKS, type-3 polyketide synthase.

Type	Most similar known cluster		Similarity
T1PKS, NRPS-like	Borrelidin	Polyketide: Modular Type I	11%
T2PKS, T1PKS	Spore Pigment	Polyketide	83%
NRPS-like, PKS-like, terpene	Phenalinolactone A	Terpene + Saccharide: Hybrid/tailoring	94%
NRPS, RRE-containing			
siderophore, NRPS	Friluimicin A /Friluimicin B / Friluimicin C / Friluimicin D	NRP	21%
NRPS			
RiPP-like			
Terpene	Geosmin	Terpene	100%
NRPS	Kirromycin	NRP + Polyketide:Modular type I + Polyketide:Trans-AT type I	13%
NRPS	Diisonitrile Antibiotic SF2768	NRP	66%
Other, T1PKS, PKS-like	Tetronasin	Polyketide	3%
T3PKS	Herboxidiene	Polyketide	7%
T1PKS	Tetronasin	Polyketide	3%
T1PKS, NRPS	Chlorothricin / Deschlorothricin	Polyketide:Modular type I + Polyketide:Iterative type I + Saccharide:Oligosaccharide	13%
NRPS, ectoine	Showdomycin	Other	23%
other	Actinomycin D	NRP	71%
lanthipeptide-class-iii			
NRPS,lanthipeptide-class-ii	Bleomycin	NRP:Glycopeptide + Polyketide:Modular type I + Saccharide:Hybrid/tailoring	9%
Terpene	Julichrome Q3-3 / Julichrome Q3-5	Polyketide	25%
ectoine	Ectoine	Other	100%
T1PKS, NRPS-like, transAT-PKS-like	Cinnabaramide A	NRP + Polyketide:Modular type I	18%
NRPS-like,NRPS,T1PKS	Bleomycin	NRP:Glycopeptide + Polyketide:Modular type I + Saccharide:Hybrid/tailoring	6%
other,lanthipeptide-class-iv	A-503083 A / A-503083 B / A-503083 E / A-503083 F	NRP	5%
RiPP-like	Informatipeptin	RiPP:Lanthipeptide	28%
terpene			
NRPS,NAPAA	Stenothricin	NRP:Cyclic depsipeptide	18%
NRPS	Mirubactin	NRP	64%
T1PKS, siderophore	Kinamycin	Polyketide	22%
Terpene, thiopeptide	Hopene	Terpene	92%
NRPS-like, betalactone	Nucleocidin	Other	8%
T1PKS	Filipin	Polyketide	92%
NRPS	Cyclothiazomycin	RiPP:Thiopeptide	9%
NRPS			
terpene	Ebelactone	Polyketide	5%
NRPS, NRPS-like			
terpene			
Siderophore	desferrioxamine	Other	66%
melanin	Melanin	Other	60%
NRPS			

*Streptomyces* sp. ADLamb9 has the genetic potential to produce structurally diverse metabolites, including siderophores, terpenes, nonribosomal peptides, polyketides, pigments, ribosomally synthesized and post-translationally modified peptide products (RiPPs). Several gene clusters were 100% identical to known gene clusters, such as those involved in making the osmoprotectant ectoine (100%) [12,13]. This genome also has the potential to produce a molecule similar to the antifungal agent filipin (92%) [14], as well as other terpenes, nonribosomal peptides, and polyketides. Additionally, several gene clusters show similarities to pharmacologically relevant compounds, including actinomycin D (71%) [15].

**Fig. 6:**
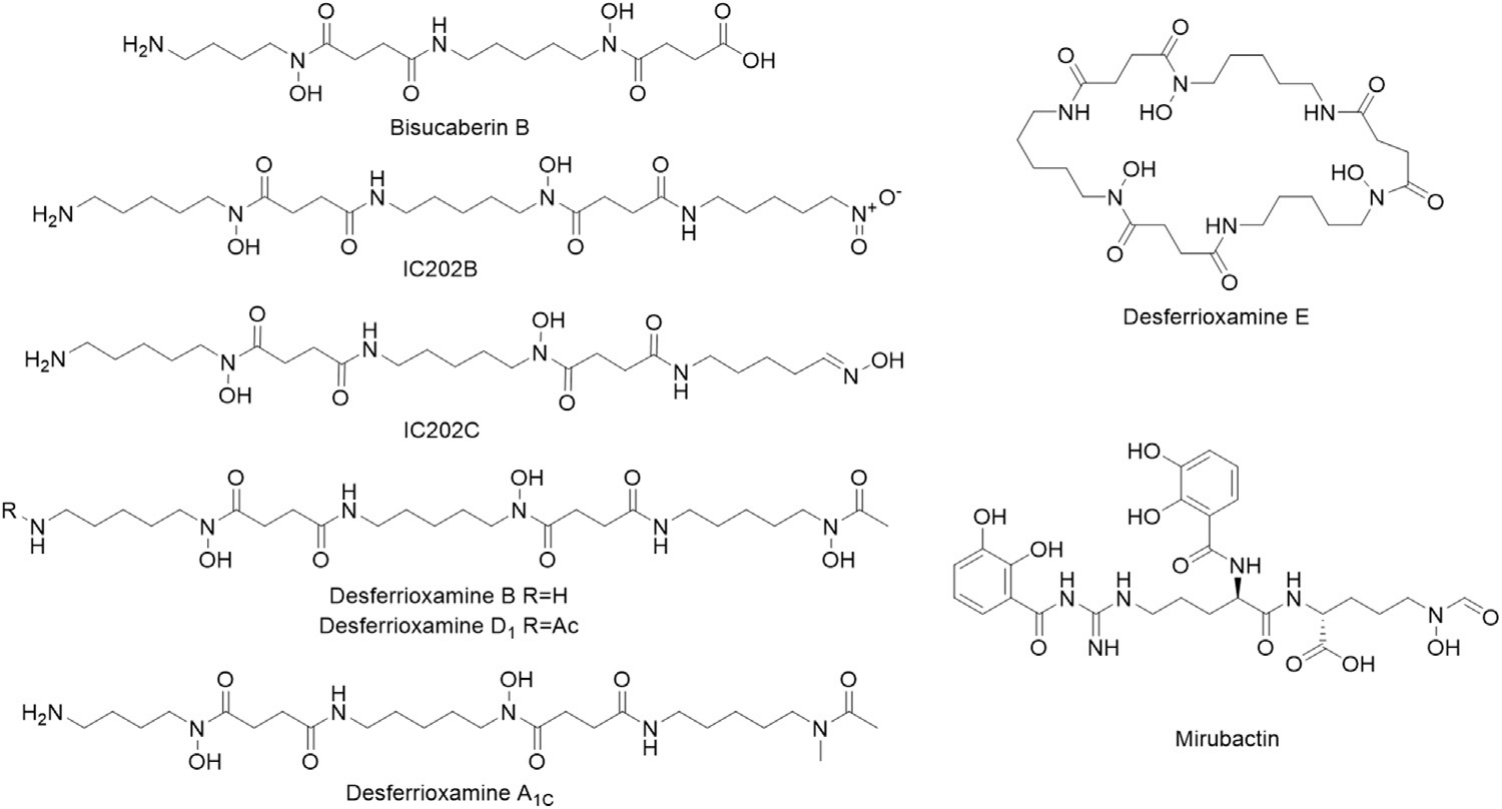
Chemical structures of identified siderophores in *Streptomyces* sp. ADLamb9 cultures.

To further explore siderophore production, *Streptomyces* sp. ADLamb9 was grown in microcultures under various growth conditions. Different cerium edetate concentrations were used to evaluate dose-dependent effects on secondary metabolite production. The metabolomic analysis of *Streptomyces* sp. ADLamb9 (Table 3) revealed a diverse profile of siderophores, including the hydroxamates bisucaberin B and desferrioxamines, as well as the dipeptide mirubactin (Fig. 6).

**Table 3:** Identified siderophores and their properties in *Streptomyces* sp. ADLamb9 Metabolomic Analysis.

Compound	Formula	Retention time	M+H	Theoretical M+H
Bisucaberin B	C_18_H_34_N_4_O_7_	3.50	419.2495	419.2506
IC202C	C_23_H_45_N_6_O_7_	3.83	517.3342	517.3350
IC202B	C_23_H_44_N_6_O_8_	4.34	533.3292	533.3299
Desferrioxamine B	C_25_H_48_N_6_O_8_	3.71	561.3604	561.3612
Desferrioxamine E	C_27_H_48_N_6_O_9_	4.75	601.3561	601.3561
Desferrioxamine D_1_	C_27_H_50_N_6_O_9_	4.62	603.3707	603.3718
Mirubactin	C_26_H_32_N_6_O_11_	4.50	605.2207	605.2207
Desferrioxamine A_1C_	C_24_H_46_N_6_O_8_	3.82	547.3447	547.3455

**Fig. 7:**
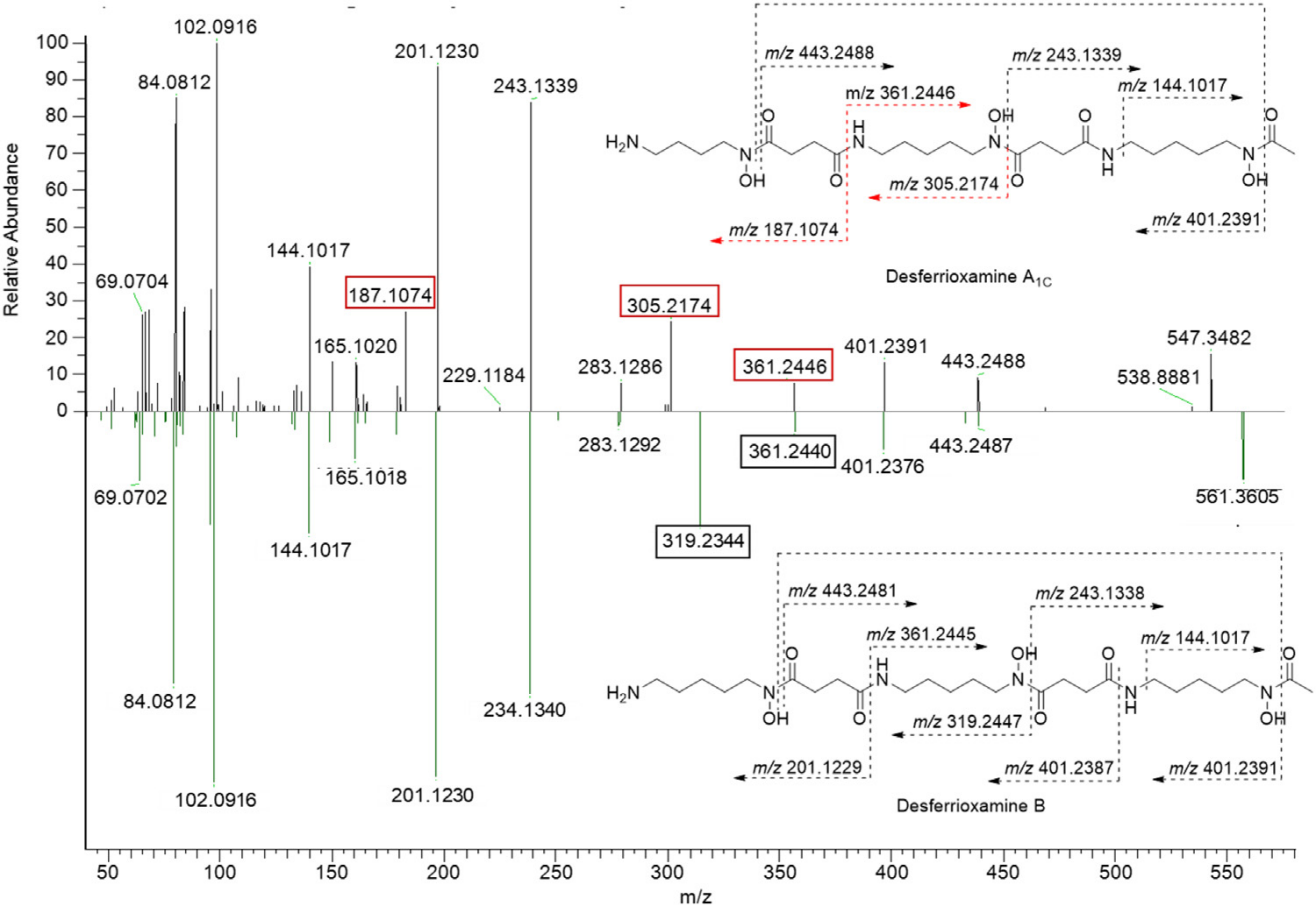
Mirrored MS/MS spectra of desferrioxamine A_1C_ (top) and desferrioxamine B (bottom) with key fragments for the elucidation of the structure of desferrioxamine A_1C_ indicated.

A new compound desferrioxamine A_1C_ was also detected in *Streptomyces* sp. ADLamb9 extracts (Fig. 7). The putative structure of desferrioxamine A_1C_ was assigned through fragmentation pattern analysis with the appearance of new peaks at 187.1074 and 305.2174 *m/z* being shifted by approximately -14.014 *m/z* from the corresponding fragments in desferrioxamine B. The retention of the 361.2446 *m/z* peak in both spectra were key to verifying the position of cadaverine replacement by putrescine occurring distal to the terminal acetate moiety (Fig. 7). Furthermore, IC202B, previously associated with *Streptoallotechius sp.* 1454-19, a rare and uncommon nitro compound, was also detected [16].

**Fig. 8:**
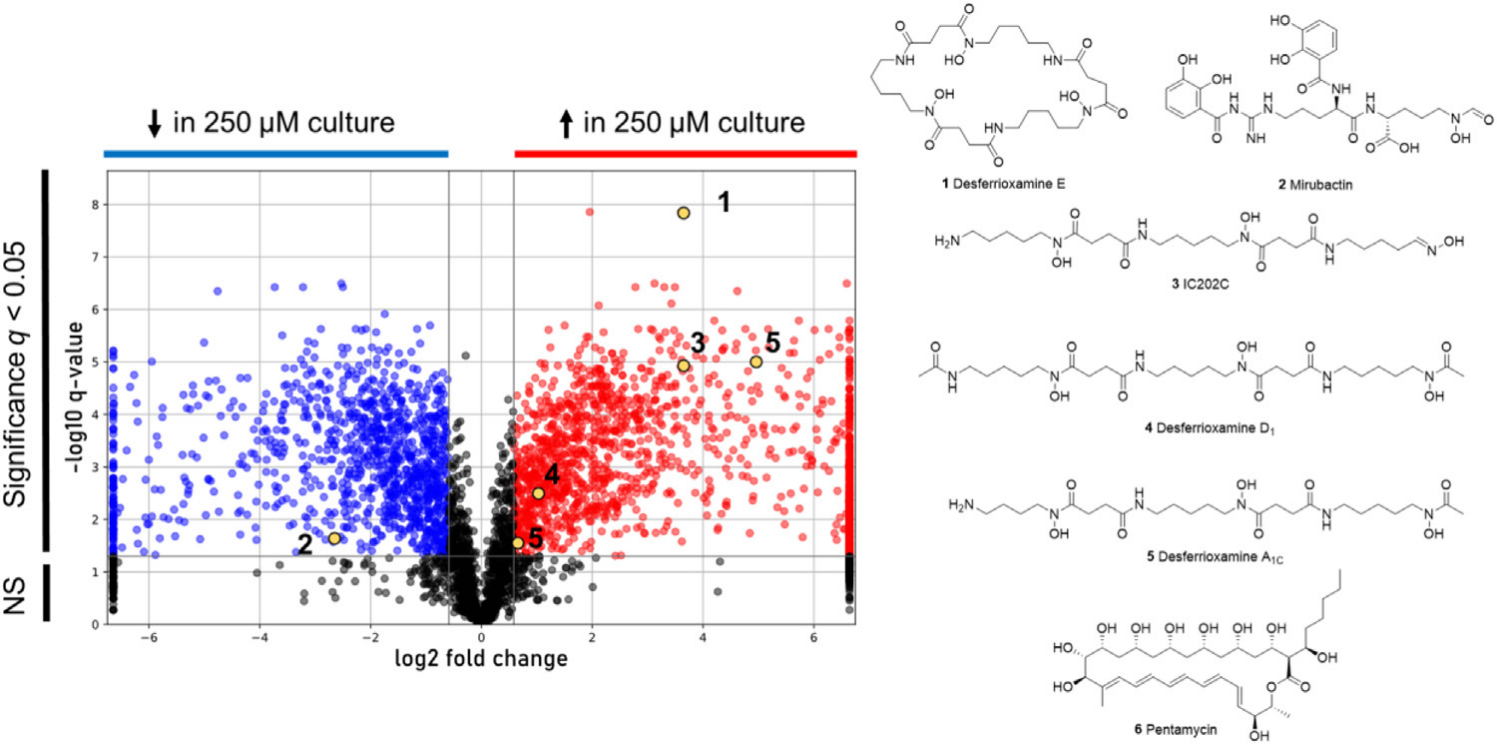
Volcano plot of differentially expressed features in *Streptomyces* sp. ADLamb9 cultures. Statistically significant features that are upregulated in the presence of cerium are shown in red, whereas those that are downregulated are in blue. The chemical structures of key upregulated metabolites are shown on the right.

Cerium is a rare earth element that has been reported to impact secondary metabolism [4]. The volcano plot in Fig. 8 shows that cerium elicitation significantly induced the accumulation of a suite of structurally diverse hydroxamate siderophores, including desferrioxamines B, E, D_1,_ A_1C,_ as well as IC202C. Conversely, cerium significantly reduced levels of the catecholate mirubactin, selectively inducing the accumulation of hydroxamate desferrioxamines over the catecholate mirubactin.

## 4 Experimental Design, Materials and Methods

### 4.1 Sample collection and microbial isolation

In September of 2024, soil samples of *L. dentata* were collected in sterile tubes using sanitized nitrile gloves from the Show House of the Botanic Garden at Smith College in Northampton, MA. Soil samples (0.5 grams) were dried at 50°C for one hour, to sterilize, eliminate contaminants, and selectively promote spore-forming bacteria. Soil was reweighed, and mixed with 4.2 mL of autoclaved water. This mixture was stirred at 100 rpm for one hour and subsequently centrifuged at 6000 × *g* for three minutes at room temperature. To enumerate bacteria in soil samples and isolate pure strains of single species, 10-fold serial dilutions were performed through successive logarithmic reductions. A 100 µL-aliquot from each dilution was plated on GYM agar medium containing glucose (2.1 g/L), yeast extract (2 g/L), malt extract (1.1 g/L), and Bacto Dehydrated Agar (BD Biosciences; Franklin Park, New Jersey) (10.1 g/L). The media was supplemented with nalidixic acid (20 mg/L) and cycloheximide (80 mg/L), samples were spread, and plates were incubated at 37°C for one week.

Sporulating actinobacterial isolates were selected and streaked onto Difco marine agar plates (BD Biosciences, Franklin Park, NJ; 55.1 g/L) as well as GYM agar plates supplemented with nalidixic acid (20 mg/L) and cycloheximide (80 mg/L), to inhibit non-target organisms. A four-quadrant streaking technique was employed to isolate individual colonies. Plates were incubated at 37°C for seven days to promote growth, then stored at 4°C.

### 4.2 Confirmation of siderophore-producing bacteria

Siderophore production by the bacterial isolate was assessed using the method developed by Schwyn and Neilands [17]. Agar plates inoculated with bacterial isolates were incubated at 37°C temperature for at least one week, overlayed with agar containing CAS dye, and then incubated at room temperature. Plates were monitored over a one-week period for the appearance of orange halos, which indicated siderophore-producing isolates.

### 4.3 Assessment of antibacterial and antifungal activity

To evaluate the antibacterial activity of selected sporulating actinobacterial isolates, *Bacillus subtilis* was uniformly streaked across a GYM agar plate, and the isolate was then added to quadrants on the plate. The same technique was applied for antifungal assays using *Aspergillus niger* and *A. flavus*. Plates were incubated at 37°C for one week, and antibacterial and antifungal activities were assessed by observing the zones of inhibition around each isolate.

### 4.4 Illumina sequencing of ADLamb9

#### 4.4.1 DNA extraction, library preparation, and sequencing

DNA extraction from selected isolates was performed using the ZymoBIOMICS DNA Microprep Kit (Zymo Research, Irvine, CA) according to the manufacturer’s instructions. The NEBNext Ultra Express FS Library Prep Kit (New England Biolabs, Ipswich, MA, USA) was utilized for library preparation of the extracted DNA. The concentration of the prepared library was determined using a Qubit 4 Fluorometer (Thermo Fisher Scientific, Waltham, MA, USA). Quality control of the prepared libraries was conducted using the Fragment Analyzer 5200 (Santa Clara, CA, USA) to ensure that the libraries met the necessary quality standards. Sequencing was then performed on the Illumina MiSeq platform in the Center for Molecular Biology at Smith College (Northampton, MA) using the MiSeq Reagent Kit v3 (600 cycles).

### 4.5 Genome assembly and bioinformatics

Paired-end sequencing data were processed using Trimmomatic (version 0.39) [16] to remove low-quality reads and adapters, followed by quality control with FastQC (version 0.12.1) [9] to ensure high-quality sequences. The cleaned reads were assembled into contigs using Unicycler (version 21.20.50), with assembly quality assessed by Quast (version 21.46.33) [5,6]. BUSCOv5 was used to estimate genome completeness and redundancy of genomic data [5]. Contamination of the assembled genome was evaluated by the NCBI Foreign Contamination Screen [6] Secondary metabolite biosynthesis gene clusters were identified using antiSMASH [11]. To confirm the taxonomic identification, Sanger sequencing was also performed on the 16S rDNA gene amplified by the polymerase chain reaction (PCR) using the following primers: [27F primer- 5’- AGAGTTTGATCMTGGCTCAG-’3; 1492R primer- 5’- CGGTTACCTTGTTACGACTT]. PCR was performed using the following thermocycling parameters: 94°C for 30 s, followed by 34 cycles of 94°C for 30 s, 50°C for 60 s, 68°C for 2 min, and a final extension at 68°C for 5 min. For taxonomic identification and phylogenetic analysis, the genome assembly was uploaded to the TYGS platform, where a GBDP-based phylogenetic tree was generated [8]. The draft genome of *Streptomyces* sp. ADLamb9 was also analyzed by Rapid Annotation using Subsystems Technology (RAST) [10].

### 4.6 Microscale cultures for dose-response and LC-MS/MS metabolomics studies

ADLamb9 spores were used to inoculate 10 mL of GYM media. A 2.5 mM cerium stock solution was prepared by combining equal volumes of 5 mM aqueous stocks of CeCl_3_·7H_2_O and disodium ethylenediaminetetraacetic acid (EDTA) solutions (Sigma-Aldrich, St. Louis, MO, USA). The following cultures were prepared in 96-well plates in triplicate without shaking at 30°C for 7 days: 180 µL of GYM liquid media and 20 µL inoculant; 180 µL GYM liquid media, 20 µL prepared inoculant, and 20 µL of 2.5 mM sodium cerium edetate stock solution (to promote secondary metabolite production); and a blank with 200 µL GYM liquid media. After one week, 200 µL from each well was added to 200 µL of LC-MS grade methanol, mixed, sonicated for 60 seconds, and centrifuged for 5 minutes at 6000 x *g* at room temperature before LC-MS analysis.

### 4.7 Liquid chromatography-mass spectrometry

For LC-MS analysis, a Thermo Scientific Q-Exactive HF-X Hybrid Quadrupole-Orbitrap mass spectrometer (Waltham, MA) was coupled with a Vanquish Horizon Ultra High-Performance Liquid Chromatography (UHPLC) system and a VH-D10-A UV detector. A Waters HSS T3 C18 column (1.8 µm, 2.1 × 150 mm) was used for separation (Waters, Milford, MA). A sample volume of 2 µL was injected onto the column at a flow rate of 0.5 mL/min. The column temperature was maintained at 40°C throughout the analysis. The UHPLC gradient was as follows: 2% acetonitrile and 98% water containing 0.1% formic acid for the first 1 minute, then increasing from 2–40% acetonitrile over 4 minutes, 40–98% over the next 3 minutes, 98–2% over 0.2 minutes, and finally 2% for the last 2 minutes. UV-vis data were collected across four wavelength ranges: 200-300, 300-400, 400-500, and 500-600 nm. Electrospray ionization (ESI) was performed using the following settings for probe position D: 40 units for sheath gas flow, 8 units for auxiliary gas flow, 1 unit for sweep gas flow, 3.5 kV spray voltage, 380°C capillary temperature, 50 for the radiofrequency (RF) funnel level, and 350°C for the auxiliary gas temperature. A Top 5 data-dependent acquisition (DDA) method was employed with MS1 scans at a resolution of 60,000 and MS2 scans at a resolution of 15,000 over a mass-to-charge (*m/z*) range of 150-2,000.

### 4.8 Data analysis

Targeted analysis was performed using Thermo Freestyle 1.8 SP2. All annotations were confirmed to match NIST 23 and/or MS-DIAL reference spectral libraries [18,19] Raw data were processed using MS-DIAL v5.5.241113 with default parameters, including profile data for both MS1 and MS2 scans, and selection of [M+H] and [M+Na] adducts [18]. A minimum peak intensity threshold of 3e5 was set, and the peak lists were analyzed with MPACT v1.00 r24.11.17 [4]. The data were filtered and visualized using the default settings in MPACT [4].

## Limitations

Several limitations were encountered during the data collection and experimental processes. Illumina sequencing generates vast amounts of data. However, de novo genome assemblies produced using this approach are often incomplete, increasing the risk of annotation errors. These issues were addressed through resequencing, which aimed to improve the overall quality of the genome. These limitations may have influenced the accuracy and completeness of the results, particularly concerning antibacterial activity and genomic analysis.

## Ethics Statement

The authors have read and followed the ethical requirements for publication in Data in Brief. The current work does not involve human subjects, animal experiments, or any data collected from social media platforms.

## CRediT Author Statement

**Adline Daly:** Investigation, Data curation, Visualization, Writing- Original draft preparation. **Robert Samples:** Conceptualization, Methodology, Formal analysis, Writing-review & editing, Visualization, Investigation, Data curation **Riccardo Racicot:** Methodology, Investigation, Data curation, Formal analysis, Validation. **Lesley-Ann Giddings:** Project Administration, Funding Acquisition, Conceptualization, Methodology, Writing-review & editing.

## Data Availability

NCBIStreptomyces sp. Adlamb9 Genome sequencing (Original data).
